# EFFICACY OF FULL-ENDOSCOPIC INTERLAMINAR AND TRANSFORAMINAL DISCECTOMY FOR LUMBER DISC HERNIATION

**DOI:** 10.1590/1413-785220233105e263326

**Published:** 2023-10-23

**Authors:** GUOQIANG ZHANG, XUEHU XIE, NING LIU

**Affiliations:** 1 Capital Medical University, Beijing Friendship Hospital, Department of Orthopedics, Beijing, China

**Keywords:** Lumbar Vertebrae, Intervertebral Disc Displacement, Endoscopic Surgical Procedures, Discectomy, Fluoroscopy., Vértebras Lombares, Deslocamento do Disco Intervertebral, Procedimentos Cirúrgicos Endoscópicos, Discotomia, Fluoroscopia.

## Abstract

A previous study has reported the therapeutic effects of interlaminar/transforaminal approaches under full-endoscopic visualization to treat L5-S1 lumber disc herniation (LDH). However, the comparison of interlaminar/transforaminal approaches to treat other segments of LDH remains unclear. Objective: To evaluate the clinical efficacy of full-endoscopic interlaminar and transforaminal lumbar discectomy to treat LDH. Methods: A total of 93 patients with LDH who underwent fully-endoscopic lumbar interlaminar/transforaminal discectomy were retrospectively collected. Patients were divided into a Transforaminal group (n=41) and an Interlaminar group (n=52). Clinical efficacy was evaluated by visual analogue scale (VAS), the Oswestry disability index (ODI), and the modified MacNab scoring system. Results: Of the 93 patients, involving segments in LDH referred to L_3-4_, L_4-5_, and L_5-S1_. The fluoroscopy times in the Interlaminar group were smaller than that of the Transforaminal group. We found no obvious significances between the Transforaminal and Interlaminar groups regarding operation time, incision length, postoperative landing time, hospitalization, and incision healing time. Postoperative VAS and ODI scores notably improved at follow-up. Besides, almost 90% LDH patients achieved excellent/good outcomes. Conclusion: The full-endoscopic visualization technique via interlaminar and transforaminal approaches safely and effectively treat LDH. *Level of Evidence III, Retrospective Study.*

## RESUMO

Um estudo anterior relatou os efeitos terapêuticos das abordagens interlaminar/transforaminal sob visualização totalmente endoscópica para tratar a hérnia de disco lombar (HDL) L5-S1. No entanto, a comparação das abordagens interlaminar/transforaminal para o tratamento de outros segmentos de HDL permanece pouco clara. Objetivo: Avaliar a eficácia clínica da discectomia lombar interlaminar e transforaminal totalmente endoscópica no tratamento da HDL. Métodos: Foram recolhidos retrospetivamente 93 pacientes com HDL submetidos a discectomia lombar interlaminar/transforaminal totalmente endoscópica. Os pacientes foram divididos em um grupo transforaminal (n=41) e um grupo interlaminar (n=52). A eficácia clínica foi avaliada através da escala visual analógica (EVA), do índice Oswestry de incapacidade (ODI) e do sistema de pontuação de MacNab modificado. Resultados: Dos 93 pacientes, os segmentos envolvidos na HDL referiam-se a L_3-4_, L_4-5_ e L_5-S1_. Os tempos de fluoroscopia no grupo Interlaminar foram menores do que no grupo Transforaminal. Não encontramos significâncias óbvias entre os grupos Transforaminal e Interlaminar em relação ao tempo de operação, comprimento da incisão, tempo de pós-operatório, hospitalização e tempo de cicatrização da incisão. As pontuações EVA e ODI pós-operatórias melhoraram notavelmente no acompanhamento. Além disso, quase 90% dos pacientes com HDL obtiveram resultados excelentes/bons. Conclusão: A técnica de visualização totalmente endoscópica através de abordagens interlaminar e transforaminal trata a HDL de forma segura e eficaz. *Nível de Evidência III, Estudo Retrospectivo.*

## INTRODUCTION

Lumber disc herniation (LDH) is a common orthopedic disease, especially stemming from the degeneration and injury of lumbar intervertebral discs and mainly manifesting itself as low back pain.^1^ LDH generally requires surgical treatment. Although fenestration discectomy (FD) has a definite efficacy, it may include disadvantages such as great trauma and long postoperative recovery time.^2^ Thus, spine surgeons have introduced minimally invasive procedures, such as microsurgical discectomy, microendoscopic discectomy, and so on.^3^ ^), (^ ^4^ ^), (^ ^5^ Percutaneous endoscopic lumbar discectomy (PELD) has received greater attention as another minimally invasive spinal surgery due to its small incisions, fast recovery, short hospital stay, and clinical efficacy.^6^ ^), (^ ^7^ However, transforaminal PELD (PETD) offers difficulties when performed in the high iliac crest and narrow foramen at L5-S1.^8^ Thus, improving the safety of the operation and reducing patients’ radiation exposure to ensure the efficacy of the procedure has become another development direction.

Today, full-endoscopic discectomy is performed under high-quality visualization to reduce fluoroscopy times.^9^ ^), (^ ^10^ Correspondingly, Aydın and Bolat^11^ have confirmed that full-endoscopic lumbar discectomies produces beneficial outcomes. Another similar study has stated that full endoscopic lumbar discectomies are effective for patients with all types of LDH, including severely difficult and extremely difficult cases.^12^ Although full-endoscopic visualization can be used to conduct interlaminar and transforaminal discectomies, only one report has compared which approach better treats L5-S1 disc herniation.^13^ However, the comparison of interlaminar and transforaminal approaches to treat other segments of LDH remains unclear.

This study investigated the clinical efficacy of discectomy by full-endoscopic visualization via the interlaminar and transforaminal approaches to treat LDH at L5-S1 and other levels.

## METHODS

### Patients

A total of 93 patients with LDH who underwent fully-endoscopic interlaminar/transforaminal lumbar discectomies from June 2019 to December 2020 were retrospectively collected in this study. Of these, 57 cases had central disc herniation and 36, paracentral disc herniation. Patients who 1) were diagnosed with LDH; 2) had central or paracentral protrusion; 3) had received conservative treatments for more than three months with no significant effect; 4) had no severe ossification with or without disc calcification; and 5) had complete follow-up data were included in this study. Patients who 1) had symptoms and signs that were inconsistent with imaging findings; 2) had recurrent LDH; 3) had LDH and lumbar instability, lumbar spondylolisthesis, lumbar infection, lumbar tumor, mental disorder, etc., were excluded from this research.

All LDH patients were operated under the full-endoscopic visualization surgical system (Jaime, Karlsruhe, Germany). According to this surgical approach, patients who were treated with fully-endoscopic transforaminal visualization were defined as the Transforaminal group (n=41), whereas patients who received the interlaminar approach were defined as the Interlaminar group (n=52). This study was approved by the Ethics Committee of the Beijing Friendship Hospital, Capital Medical University (2021-P2-385-01). Written informed consent was given by all participants.

### Surgery technique

**Figure 1:**
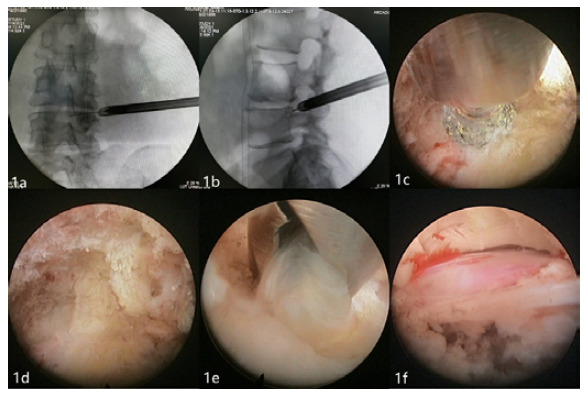
Operation procedure for the full-endoscopic visualization technique via the transforaminal approach. Intraoperative anteroposterior and lateral radiographs confirmed the intervertebral gap and foramina (a, b); The ventral superior articular process was shaped by a power drill (c); Ligamentum flavum and posterior edge of the intervertebral disc (d); Removal of the prominent nucleus pulposus (e); Exploration of nerve root relaxation with no nucleus pulposus left (f).

In the Transforaminal group, patients were positioned prone and locally anesthetized. Under the guidance of a C-arm X-ray detector, the distance between the median line and the lesion segment was opened 8-14 cm laterally as a simulated puncture point. After routine disinfection, a no.-18 puncture needle was inserted into patients’ intervertebral foramen, followed by a guide wire. An 8-mm incision was made in patients’ skin with the puncture point as the midpoint, and guide rods were placed in steps to expand the surrounding soft tissue. Following the introduction of a working cannula and the endoscopic surgical system, foraminoplasty was performed with a power drill under endoscopic viewing. Subsequently, the protruding nucleus pulposus tissue was completely removed until the nerve roots were exposed. When the dura mater and nerve roots fluctuated well, patients’ fibrous annulus was corrugated with a plasma radiofrequency, ending the operation (Figure 1).

**Figure 2:**
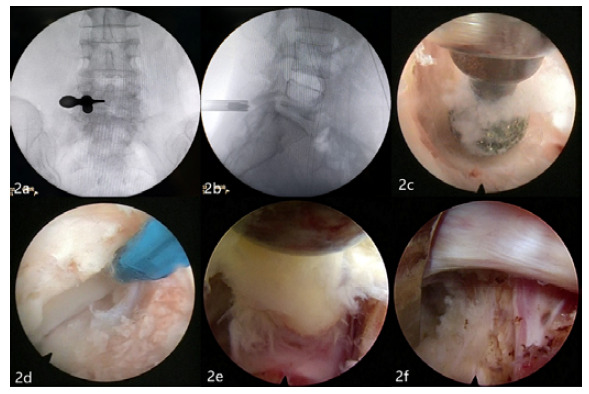
Procedures for full-endoscopic visualization via the interlaminar approach. Intraoperative anteroposterior and lateral radiographs confirmed the intervertebral gap and interlaminar space (a, b); Fenestration of interlaminar spaces on the affected side was achieved by a power drill (c); The insertion of the ligamentum flavum is exposed from the inner edge of the superior articular process (d); The outer edge of the autonomic nerve root was exposed inwardly and the prominent nucleus pulposus tissue was removed (e); Exploration of nerve root relaxation with no nucleus pulposus left (f).

Interlaminar group patients were positioned prone under general anesthesia. The vertebral pedicle inner wall line of the surgical segment and the intersection of the upper vertebral body lower margin line were chosen as puncture points. After routine disinfection, a no.-18 puncture needle was inserted into the outer edge of patients’ interlaminar space. An 8-mm incision was made in patients’ skin and guide rods were placed in steps to expand the surrounding soft tissue. After the working cannula and endoscopic surgical system were introduced, interlaminar fenestration was performed by a power drill under endoscopic viewing. Part of the ligamentum flavum was removed to expose the nerve root and the protruding nucleus pulposus tissue was completely excised. When the dura mater and nerve roots fluctuated well, patients’ fibrous annulus was corrugated by plasma radiofrequency, ending the operation (Figure 2).

### Postoperative management

Routine analgesic and neurotrophic drugs were postoperatively given to patients for three days.

One day after surgery, patients wore a waist circumference and were moved on the ground. Then, three days after the procedures, functional exercises were postoperatively conducted, including lower limb and lumbar back muscles exercises. Strenuous activities were avoided for one month after operation.

### Postoperative evaluation

Operation time, fluoroscopy number, incision length, postoperative landing time, hospitalization time, incision healing time, and complications were recorded. The visual analogue scale (VAS) was used to evaluate patients’ pain symptoms before and after surgery. Volunteers’ ability of daily living was assessed by the Oswestry disability index (ODI). All patients were followed up for at least 12 months after surgery. The modified MacNab scoring system was utilized to evaluate surgery efficacy.

### Statistical analysis

Data are shown as mean±standard deviation (SD) and analyzed using SPSS 20.0. Data were enumerated using the chi-squared test. Perioperative data were compared using Student’s t-test. The significances during follow-up (including VAS and ODI) were analyzed by two-away ANOVA followed by the Bonferroni test. A p<0.05 was deemed as a significant difference.

## RESULTS

### General characteristics

Table 1 shows no obvious significances in gender, age, BMI, follow-up time, waist and leg VAS scores, and ODI between the two groups (p>0.05). However, the operation segments in the Interlaminar group obviously differed from those in the Transforaminal group (p<0.05).

**Table 1:** Baseline characteristics.

	Transforaminal group (n=41)	Interlaminar group (n=52)	t/χ^2^ value	p-value
Age (years)	53.44±14.09	49.69±14.207	1.267	0.208
BMI (kg/m^2^)	23.36±1.47	23.45±1.38	-0.312	0.756
Operation segments (n)			50.02	0.000
L3-4	3	-	-	-
L4-5	37	13	-	-
L5-S1	1	39	-	-
Waist VAS score	5.68±1.01	5.58±1.00	0.506	0.614
Leg VAS score	6.88±1.01	6.85±1.02	0.151	0.880
ODI (%)	39.71±9.32	39.67±9.11	0.018	0.986
Follow-up (months)	20.88±6.03	21.21±5.04	-4.033	0.127

BMI: body mass index; VAS: visual analogue scale; ODI: Oswestry disability index.

### Operative technique and perioperative outcome

Patients of both groups received successful operations. The fluoroscopy times in the Transforaminal group (12.27±1.07) were longer than in the Interlaminar group (8.02±0.78) (p=0.000). Moreover, we found no obvious significances between the Transforaminal and Interlaminar groups regarding operation time, incision length, postoperative landing time, hospitalization time, and incision healing time (p>0.05) (Table 2).

**Table 2:** Comparison of perioperative data between the two groups.

	Transforaminal group (n=41)	Interlaminar group (n=52)	t value	p-value
Operation time (min)	150.15±37.47	151.54±37.58	-0.178	0.859
Incision length (mm)	10.05±0.87	10.04±0.91	0.056	0.956
Fluoroscopy times (n)	12.27±1.07	8.02±0.78	21.31	0.000
Postoperative landing time (day)	1.12±0.40	1.42±1.24	-1.644	0.105
Hospitalization time (day)	6.76±0.44	6.75±0.44	0.067	0.947
Incision healing time (day)	13.37±0.80	13.42±0.72	-0.362	0.718

### Clinical outcomes

Follow-up showed no recurrence in both groups and some patients had pain and activity limitation that were relieved by painkillers and physical therapy. Additionally, we found no serious complications such as intervertebral space infections, lower limb thrombosis, dural sac ruptures, and vascular injuries.

Table 3 shows that VAS scores and ODI failed to significantly differ between both groups, whereas we found an obvious time effect. We observed no interactions between times and the groups.

**Table 3:** Comparison of visual analogue scale and Oswestry disability index between both groups.

	Time	Transforaminal group (n=41)	Interlaminar group (n=52)	Between-group effect	Within-group effect	Interaction
Waist VAS score	Pre-operation	5.68±1.011	5.58±0.997	F=0.000, P=0.993	F=392.241, P<0.001	F=0.300, P=0.753
	Postoperative 3 months	2.95±0.999*	3.00±1.103*			
	Postoperative 6 months	2.85±0.963*	2.83±1.098*			
	Last follow-up	2.27±0.837*	2.35±1.008*			
Leg VAS score	Pre-operation	6.88±1.005	6.85±1.017	F=0.000, P=0.988	F=910.141, P<0.001	F=0.034, P=0.910
	Postoperative 3 months	2.56±0.976*	2.58±1.091*			
	Postoperative 6 months	2.39±0.919*	2.38±0.973*			
	Last follow-up	2.22±0.725*	2.25±0.837*			
ODI (%)	Pre-operation	39.71±9.320	39.67±9.113	F=0.036, P=0.849	F=193.316, P<0.001	F=0.113, P=0.890
	Postoperative 3 months	28.05±7.096	28.06±7.524			
	Postoperative 6 month	24.34±7.445*	23.44±8.278*			
	Last follow-up	18.24±7.562*	18.21±7.920*			

VAS: visual analogue scale; ODI: Oswestry disability index.

Regarding within-group differences, VAS scores of waist and leg in two groups significantly decreased at three months after the procedures and then tended to stable (p<0.05). However, ODI in two groups decreased six months after the operations and the last follow-up (p<0.05).

### Efficacy evaluation

According to the modified MacNab evaluation criteria, 90.24% of the Transforaminal group showed excellent and good outcomes. Briefly, 31 cases were excellent, 6 good, 3 fair, and 1 poor.

**Figure 3:**
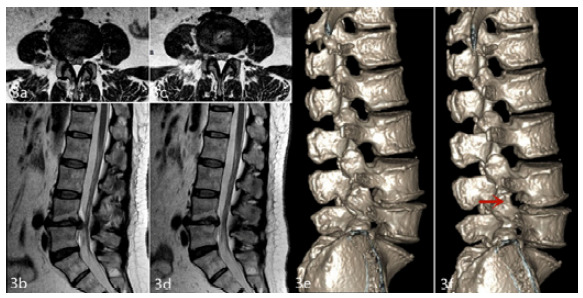
Representative lumber disc herniation case pre- and post-operation (L4-5, right paracentral type, female). Preoperative lumbar MRI showed that the L4-5 intervertebral disc was herniated to the right and that the L5 nerve root and dural sac were compressed (a, b); 12 months after the operation, lumbar MRI showed that the herniated intervertebral disc tissue had disappeared and that the right L5 nerve root and dural sac compression were relieved (c, d); Comparison of a three-dimensional CT reconstruction of the lumbar spine before and 12 months after the operation (the arrow indicates the L4-5 intervertebral foramen post-operation enlargement) (e, f).

**Figure 4:**
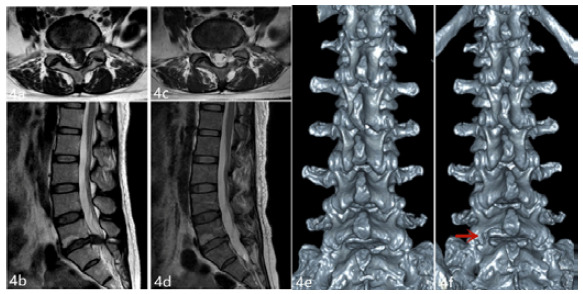
Representative lumber disc herniation case pre- and post-operation (L5-S1, left paracentral type, female). Preoperative lumbar MRI showed that the L5-S1 intervertebral disc was herniated to the left and that the S1 nerve root and dural sac were compressed (a, b); 12 months after the operation, lumbar MRI showed that the herniated intervertebral disc tissue had disappeared and that the left S1 nerve root and dural sac compression was relieved (c, d); Comparison of a three-dimensional CT reconstruction of the lumbar spine before the operation and 12 months after it (the arrow indicates the increase of the L5-S1 left interlaminar bone window after the operation) (e, f).

The Interlaminar group had 40 excellent cases (76.92%), six (11.54%) good cases, four (7.69%) fair cases, and two (3.85%) poor cases, showing a 88.46% excellent and good case rate. We found no statistically significant difference between both groups (χ^2^=0.320, p=0.956) (Table 4). Figures 3-4 show the representative cases.

**Table 4:** Efficacy evaluation at last follow-up based on the MacNab score system.

	Transforaminal group (n=41)	Interlaminar group (n=52)	^χ2^ **value**	p-value
Excellent (n, %)	31 (75.61)	40 (76.92)		
Good (n, %)	6 (14.63)	6 (11.54)		
Fair (n, %)	3 (7.32)	4 (7.69)		
Poor (n, %)	1 (2.44)	2 (3.85)		
Total (n, %)	41 (100)	52 (100)	0.320	0.956

## DISCUSSION

Growing evidence has shown that lumbar discectomy under the full-endoscopic technique ensures surgery safety by improving the visualization of the procedures.^14^ ^), (^ ^15^ ^), (^ ^16^ Operations generally employ two approaches: transforaminal and interlaminar accesses. Additionally, spine surgeons know full-endoscopic transforaminal discectomy better to due to its relative maturity.^17^ ^), (^ ^18^ Correspondingly, the interlaminar approach was initially applied to patients with L5-S1 disc herniation to achieve sufficient resection.^9^ However, the comparison of interlaminar and transforaminal approaches for LDH patients is yet to be fully reported. In this study, we found that both approaches under the guidance of the full-endoscopic technique achieved satisfactory efficacy.

Regarding anesthesia, a previous study has reported that full-endoscopic transforaminal and interlaminar lumbar discectomies were employed to treat L5-S1 LDH under general anesthesia.^13^ A similar study has used the two approaches to treat all types of LDH under general anesthesia.^11^ Nevertheless, Kim et al.^12^ believed that PELD surgery can be performed under local anesthesia. LDH may show anomalous lumbosacral nerve roots and cutting these nerves would cause irreversible damage.^19^ ^), (^ ^20^ In the case of nerve root variation, PETD may damage the nerve root as patients are unable to give feedback to surgeons due to the general anesthesia. In our study, Transforaminal group patients received local anesthesia, supporting its effectiveness on lumbar discectomies.^21^ ^), (^ ^22^

A previous study has reported that the average operation time of full-endoscopic lumbar discectomies via interlaminar and transforaminal approaches range from 40 to 210 min, respectively.^13^ In this study, the average operative times of both groups conformed to the aforementioned reports. Moreover, we observed no obvious difference between the two groups regarding operative times. This may stem from our inclusion of LDH patients with different involved segments. Compared with full-endoscopic technique, patients during traditional PELD undergo longer fluoroscopy, causing excessive radiation exposure.^23^ This study had fluoroscopy times ranging from 8.02 to 12.27 sec, lower in the Interlaminar group than in the Transforaminal group. A previous study has also confirmed that LDH L5-S1 patients undergoing the full-endoscopic technique via interlaminar approach undergo shorter fluoroscopies.^13^ Another study has reported that the interlaminar approach can decrease intraoperative radiation exposure.^8^ Furthermore, the ODI and VAS scores at the last follow-up significantly improved, whereas the efficacy between both groups was similar. Therefore, full-endoscopic visualization via interlaminar or transforaminal approaches can achieve good efficacy, indicating that it can reduce damage to the posterior lumbar spine under visual control.^24^

Recurrence and complications are important issues. Previous studies have reported that recurrence rates after PELD ranged from 0 to 5%.^11^ ^), (^ ^25^ ^), (^ ^26^ This study found no recurrence in either the interlaminar or transforaminal groups during follow-up. Alternatively, the type of disc herniation and anular defect may be associated with recurrence rates.^27^ In our study, a drill head with a 2.5-mm diameter under full-endoscopic technique could precisely and controllably resect bones, avoiding the postoperative “iatrogenic lumbar instability” due to the large diameter (diameter ≥7.5 mm) of bone resecting ring saws. This result agrees with a previous study that considered minimizing anular defects during operation as a protective factor.^28^ Fortunately, almost 90% of our LDH patients obtained good clinical outcomes without serious complications. Yörükoğlu et al.^29^ have analyzed complications using fully endoscopic interlaminar or transforaminal lumbar discectomies, finding that complications occur but most are resolved spontaneously. Taken together, the full-endoscopic technique via the transforaminal or interlaminar approaches can improve the treatment of LDH patients.

Although both approaches can produce good therapeutic effect, they have corresponding indications. For instance, Choi and Park^30^ have reported that the highest point of the iliac crest above the midline of the L5 pedicle will make the L5-S1 intervertebral foraminal approach ineffective. To avoid the influence of high iliac crest and other anatomical factors, Ruetten et al.^31^ reported that 97.4% of patients with L5-S1 disc herniation have received the interlaminar approach with definite effects. Correspondingly, we also preferred the interlaminar approach in L5-S1. For patients with advanced age, poor physical fitness, and cardiovascular or cerebrovascular diseases, we tend to perform PETD under greater local anesthesia as much as possible. Those findings indicate that both approaches should complement, rather than replace, each other.

This study has limitations. We conducted this retrospective study in a single center medical institution with a small sample. Additionally, our follow-up time is short. In the future, we aim to increase the size of sample and follow-up time to evaluate the clinical efficacy of this technique for LDH patients.

## CONCLUSION

The use of full endoscopic visualization technique via the transforaminal and interlaminar approaches can safely and effectively treat LDH. Importantly, patients can obtain satisfactory outcomes with less radiation exposure.
